# Impact of free cancer predisposition cascade genetic testing on uptake in Singapore

**DOI:** 10.1038/s41525-019-0096-5

**Published:** 2019-09-13

**Authors:** Eliza Courtney, Amanda Kay-Lyn Chok, Zoe Li Ting Ang, Tarryn Shaw, Shao-Tzu Li, Jeanette Yuen, Joanne Ngeow

**Affiliations:** 1 Cancer Genetics Service, Division of Medical Oncology, National Cancer Centre Singapore, Singapore, 169610 Singapore; 2 Lee Kong Chian School of Medicine, Nanyang Technological University, Singapore, 308232 Singapore

**Keywords:** Genetics research, Preventive medicine, Genetic testing, Genetic counselling, Health policy

## Abstract

Cascade testing for cancer predisposition offers a highly efficient and cost-effective method for identifying individuals at increased risk for cancer, in whom targeted interventions can often improve survival. The aim of this study was to determine the impact of free cascade testing on uptake and identify other associated factors. Demographic and clinical data were gathered prospectively for 183 probands found to have a pathogenic variant associated with cancer predisposition and their 826 first-degree relatives (FDRs). The provision of free cascade testing was significantly associated with uptake (21.6% vs 6.1%; *χ*^2^, *P* *<* 0.001). Relationship type between FDR and proband and FDR age also demonstrated significant associations, suggesting greater engagement amongst younger generations. Overall, 29.0% (53/183) of families had at least 1 FDR who underwent cascade testing. Of these families, 67.9% (36/53) had an uptake rate of at least 40.0%. Cost is a significant barrier to cascade testing uptake in Singapore. Tailored interventions targeting underrepresented groups and genetic counseling approaches supporting family communication and decision-making are necessary.

## Introduction

The increasing availability of genetic testing for cancer predisposition provides health policy makers with a significant opportunity to enhance early detection and prevention efforts.^1–3^ Hereditary causes account for approximately 5–10% of all cancer and our ability to detect such cases has improved considerably over the last two decades, with more than 400 cancer predisposition genes now described.^4,5^ Following the identification of a germline pathogenic (or likely pathogenic) variant (PV/LPV) in a gene associated with cancer predisposition in an index case presenting with disease (symptomatic proband), systematic cascade testing can then be performed in relatives who have not yet developed disease (asymptomatic) to determine their future risk. This approach is superior to family history-based risk assessments, as appropriate surveillance and/or risk-reducing strategies can be targeted towards relatives in whom the familial PV/LPV is detected and allows those without to avoid unnecessary interventions. Once identified, these individuals at increased risk can be managed appropriately before the onset of disease with the aim of reducing long-term morbidity and mortality.^6,7^

Studies have demonstrated that the degree of cascade testing uptake significantly impacts the cost-effectiveness of genetic testing programs.^8–14^ Furthermore, cascade testing has been categorized as a ‘tier 1 genomic application’ by the Centers for Disease Control and Prevention (CDC) in the USA for a number of genetic conditions, including hereditary breast and ovarian cancer syndrome (HBOC) and Lynch syndrome (LS).^1,3^ Tier 1 genomic applications are assessed by the CDC as having the potential for significant positive impact on public health and meet certain criteria for analytical validity, clinical validity, and clinical utility. Cascade testing offers a highly efficient and cost-effective method for identifying high risk individuals.^1,15^ In the case of autosomal dominant conditions, for example, a proband’s first-degree relatives (FDRs) have a 50% chance of inheriting the familial PV/LPV. In the majority of cases, this exceeds the likelihood of detecting a PV/LPV in symptomatic probands, thereby identifying more high-risk individuals with fewer genetic tests performed.^15^

Despite the evidence in support of cascade testing, there remain significant barriers to uptake and rates vary significantly.^16–23^ Of note, uptake rates reported from Asian countries have been low.^17,22,23^ Cost remains a significant barrier,^1,15,17,23^ and persists as a deterrent despite the rapid reduction in the price of genetic testing. Due to privacy legislation, communication of genetic results relies solely on proband-mediated disclosure in many countries, which is often suboptimal and complicated by a range of complex personal, social and cultural factors.^20,21,23–28^ Other frequently cited barriers to cascade testing include poor comprehension by at-risk relatives following notification, issues with access to clinical genetics services, and concerns regarding genetic discrimination.^15,22,23,29^ However, there remains a lack of ethnic and gender diversity in the literature regarding the factors that influence uptake of cascade testing.^15^ Additionally, the majority of available data concerns well-established, highly penetrant hereditary cancer syndromes, characterized by the presence of multiple related cancerous (and occasionally non-cancerous) phenotypes. Cascade testing uptake regarding genes with less well-characterized phenotypes and penetrance estimates remains under explored.

The demand for clinical genetics services in Singapore has increased dramatically,^17^ despite studies identifying persisting concerns in the community related to the potential for stigma and burden associated with heritable conditions.^23,27,30^ The cost of genetic testing for patients in Singapore remains out-of-pocket, and any available subsidies are usually sourced from finite philanthropic donations or research grants. A previous study of probands attending the Cancer Genetics Service (CGS) at the National Cancer Centre Singapore (NCCS) found that the provision of subsidies resulted in a significant increase in uptake of genetic testing.^17^ This subsidy scheme was found to be cost-effective *only* if the proportion of FDRs undergoing cascade testing exceeded 36.0%, however, uptake at the time was only 6.0%. In the time since this study was performed, fees for cascade testing are now waived for FDRs through clinical testing laboratories. For those with financial difficulties, subsidies provided by the Lee Kong Chian NCCS CGS (LKCNCCS) fund were extended to at-risk relatives of probands with an identified PV/LPV. The aims of this study were to determine the impact of free cascade testing on uptake amongst FDRs of probands seen through the NCCS CGS and secondly, explore the association of other demographic and clinical factors. The results of this study aim to inform the development of targeted interventions and health policy that may contribute to the improvement of cascade cancer genetic testing uptake amongst at-risk relatives.

## Results

### Subject characteristics

Demographic and clinical factors of the 183 eligible probands and 112 FDRs who underwent cascade testing are displayed in Table 1. The mean ages of probands and FDRs were 45.7 (95% CI: 43.6–47.9) and 38.9 (95% CI: 35.7–42.11) years, respectively. The majority of probands were female (82.0%), Chinese (74.3%) and presented for testing with the phenotype associated with the PV/LPV (symptomatic; 98.4%). Similarly, the majority of FDRs who underwent cascade testing were female (63.4%) and Chinese (71.4%), however, most did not present for testing with the phenotype associated with the familial PV/LPV (asymptomatic; 80.4%). The ethnicity proportions in both groups were reflective of the Singaporean population.^31^ More than half of probands (56.8%) have either HBOC or LS. The proportions of probands and FDRs that met criteria for LKCNCCS subsidies based on their financial status were 35.5% (65/183) and 20.5% (23/112), respectively. Of the 183 families, 1 (0.5%) had a PV/LPV in a gene associated with a X-linked condition, with the remainder (99.5%) involving genes associated with autosomal dominant conditions (some are also associated with autosomal recessive conditions, such as in the case of the mismatch repair genes and constitutional mismatch repair deficiency syndrome). Overall, the familial PV/LPV was detected in 47.3% (53/112) of tested FDRs. The mean follow-up duration was 1.69 years (95% CI: 1.53–1.85).

**Table 1:** Demographics of probands and tested FDRs

Variable	Probands (*N* = 183)	FDRs (*N* = 112)
*Age*		
Mean (CI)	45.7 (43.6–47.9)	38.9 (35.7–42.11)
*Sex*		
Male (%)	33 (18.0)	41 (36.6)
Female (%)	150 (82.0)	71 (63.4)
*Race*		
Chinese (%)	136 (74.3)	80 (71.4)
Malay (%)	28 (15.3)	20 (17.8)
Indian (%)	11 (6.0)	6 (5.4)
Others (%)	8 (4.4)	6 (5.4)
*Phenotype* ^a^		
Symptomatic (%)	180 (98.4)	22 (19.6)
Asymptomatic (%)	3 (1.6)	90 (80.4)
*Gene cohort* ^b^		
Tier 1 (%)	104 (56.8)	56 (50.0)
Syndromic (%)	55 (30.1)	47 (42.0)
Emerging evidence (%)	24 (13.1)	9 (8.0)
*LKCNCCS subsidy*		
Eligible (%)	65 (35.5)	23 (20.5)
Not eligible (%)	118 (64.5)	89 (79.5)
*Genetic result*		
PV/LPV detected (%)	183 (100.0)	53 (47.3)
PV/LPV not detected (%)	0 (0.0)	59 (52.7)

*CI* confidence interval (95%), *FDR* first-degree relative, *LKCNCCS* Lee Kong Chian National Cancer Centre Singapore Cancer Genetics Service fund, *PV/LPV* pathogenic variant/likely pathogenic variant

^a^Phenotype associated with familial PV/LPV present (symptomatic) or absent (asymptomatic) at time of testing

^b^See Supplementary Table 1 for genes included in each cohort

### Factors associated with uptake of cascade genetic testing

**Fig. 1:**
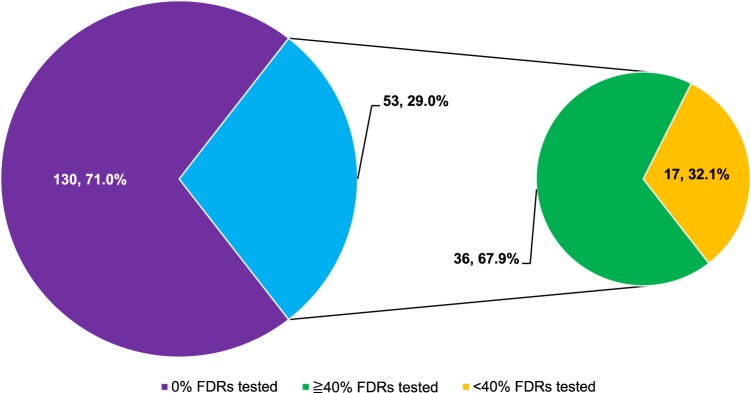
Number of families with FDRs undergoing cascade testing, by proportion of FDRs (*n*, %). The majority of families (71.0%) had no FDRs undergo cascade testing. Amongst the remaining 29.0% of families, more than two-thirds (67.9%) had ≥40.0% of FDRs undergo cascade testing. *FDR* first-degree relative

Uptake data was divided into three cohorts based on classification of gene type and is presented in Table 2, together with the total cohort. The tier 1 cohort included *BRCA1*, *BRCA2*, and the mismatch repair genes; the syndromic cohort included genes associated with other well-characterized hereditary cancer syndromes (e.g. *APC*, *PTEN*, and *TP53*); and the emerging evidence cohort included genes that are less well-characterized and have uncertain or unknown cancer penetrance estimates (see Supplementary Table 1 for genes included in each cohort). In total, 826 FDRs belonging to 183 families were included in the study. Overall, 112 (13.3%) underwent cascade testing. When separated into cohorts according to tier 1, syndromic and emerging evidence classification, the cascade testing uptake was 12.4% (56/453), 19.7% (47/238), and 6.7% (9/135), respectively. Overall, 29.0% (53/183) of families had at least one FDR who underwent cascade testing. Of these 53 families, 36 (67.9%) had at least 40.0% of FDRs undergo cascade testing (Fig. 1). The FDR uptake rate was 100.0% for 13/53 (24.5%) tested families.

**Table 2:** Proband and FDR factors associated with uptake of cascade testing amongst FDRs

Variable	Tier 1 cohort^a^ (*N* = 453)			Syndromic cohort^a^ (*N* = 238)			Emerging evidence cohort^a^ (*N* = 135)			Total cohort (*N* = 826)		
	Tested	Not tested	*P*-value	Tested	Not tested	*P*-value	Tested	Not tested	*P*-value	Tested	Not tested	*P*-value
Number												
* n* (%)	56 (12.4)	397 (87.6)	—	47 (19.7)	191 (80.3)	—	9 (6.7)	126 (93.3)	—	112 (13.3)	714 (86.4)	—
FDR factors												
*Sex*												
Male (%)	19 (8.6)	201 (91.4)	**0.019**	18 (16.2)	93 (83.8)	0.201	4 (6.5)	58 (93.5)	1.000^c^	41 (10.4)	352 (89.6)	**0.012**
Female (%)	37 (15.9)	196 (84.1)		29 (22.8)	98 (77.2)		5 (6.8)	68 (93.2)		71 (16.4)	362 (83.6)	
*Race*												
Chinese (%)	42 (13.5)	270 (86.5)	0.374	29 (16.6)	146 (83.4)	**0.006**	9 (7.7)	108 (92.3)	0.476	80 (13.2)	524 (86.8)	0.609
Malay (%)	9 (10.30)	78 (89.7)		11 (21.6)	40 (78.4)		0 (0.0)	15 (100.0)		20 (13.1)	133 (86.9)	
Indian (%)	5 (13.5)	32 (86.5)		1 (50.0)	1 (50.0)		0 (0.0)	3 (100.0)		6 (14.3)	36 (85.7)	
Others (%)	0 (0.0)	17 (100.0)		6 (60.0)	4 (40.0)		0 (0.0)	0 (0.0)		6 (22.2)	21 (77.8)	
*Age*												
Mean (CI)	47.0 (43.4–50.7)	52.1 (50.5–53.7)	**0.029** ^d^	30.1 (24.9–35.2)	43.3 (40.3–46.4)	**<** **0.001** ^d^	34.6 (27.0–42.1)	55.1 (52.5–57.8)	**<** **0.001** ^d^	38.9 (35.7–42.1)	50.3 (48.9–51.6)	**<** **0.001** ^d^
*Phenotype* ^*b*^												
Symptomatic (%)	8 (11.9)	59 (88.1)	0.910	13 (40.6)	19 (59.4)	**0.001**	1 (9.1)	10 (90.9)	0.546^c^	22 (20.0)	88 (80.0)	**0.034**
Asymptomatic (%)	48 (12.4)	338 (87.6)		34 (16.5)	172 (83.5)		8 (6.5)	116 (93.5)		90 (12.6)	626 (87.4)	
*Relationship to proband*												
Sibling (%)	31 (10.4)	268 (89.6)	**0.008**	17 (13.1)	113 (86.9)	**0.018**	1 (1.0)	95 (99.0)	**<** **0.001**	49 (9.3)	476 (90.7)	**<** **0.001**
Parent (%)	5 (8.1)	57 (91.9)		17 (27.9)	44 (72.1)		2 (11.1)	16 (88.9)		24 (17.0)	117 (83.0)	
Offspring (%)	20 (21.7)	72 (78.3)		13 (27.7)	34 (72.3)		6 (28.6)	15 (71.4)		39 (24.4)	121 (75.6)	
*Cost of testing*												
Free (%)	45 (20.1)	179 (79.9)	**<** **0.001**	36 (43.4)	47 (56.6)	**<** **0.001**	5 (5.4)	87 (94.6)	0.465^c^	86 (21.6)	313 (78.4)	**<** **0.001**
Not Free (%)	11 (4.8)	218 (95.2)		11 (7.1)	144 (92.9)		4 (9.3)	39 (90.7)		26 (6.1)	401 (93.9)	
Proband												
*Sex*												
Male (%)	3 (9.4)	29 (90.6)	0.783^c^	19 (19.4)	79 (80.6)	0.907	3 (25.0)	9 (75.0)	**0.034** ^c^	25 (17.6)	117 (82.4)	0.122
Female (%)	53 (12.6)	368 (87.4)		28 (20.0)	112 (80.0)		6 (4.9)	117 (95.1)		87 (12.7)	597 (87.3)	
*Race*												
Chinese (%)	42 (13.1)	279 (86.9)	0.449	28 (15.7)	150 (83.4)	**0.001**	9 (7.7)	108 (92.3)	0.476	79 (12.8)	537 (87.2)	0.322
Malay (%)	9 (11.5)	69 (88.5)		11 (23.4)	36 (76.6)		0 (0.0)	15 (100.0)		20 (14.3)	120 (85.7)	
Indian (%)	5 (13.5)	32 (86.5)		1 (50.0)	1 (50.0)		0 (0.0)	3 (100.0)		6 (14.3)	36 (85.7)	
Others (%)	0 (0.0)	17 (100.0)		7 (63.6)	4 (36.4)		0 (0.0)	0 (0.0)		7 (25.0)	21 (75.0)	
*Age*												
Mean (CI)	54.1 (50.8–57.5)	53.0 (51.9–54.0)	0.429^d^	27.6 (22.9–32.3)	42.5 (40.6–44.5)	**<** **0.001** ^d^	48.2 (32.7–63.8)	54.0 (52.4–55.5)	0.422^d^	42.5 (38.9–46.1)	50.3 (49.5–51.2)	**<** **0.001** ^d^
*LKCNCCS subsidy*												
Eligible (%)	16 (9.8)	147 (90.2)	0.217	26 (28.0)	67 (72.0)	**0.011**	2 (3.6)	54 (96.4)	0.305^c^	44 (14.1)	268 (85.9)	0.722
Not eligible (%)	40 (13.8)	250 (86.2)		21 (14.5)	124 (85.5)		7 (8.9)	72 (91.1)		68 (13.2)	446 (86.8)	

*CI* confidence interval (95%), *FDR* first-degree relative, *LKCNCCS* Lee Kong Chian National Cancer Centre Singapore Cancer Genetics Service fund, *PV/LPV* pathogenic variant/likely pathogenic variant

^a^See Supplementary Table 1 for genes included in each cohort

^b^Phenotype associated with familial PV/LPV present (symptomatic) or absent (asymptomatic) at time of testing

^c^Fisher’s Exact Test

^d^Independent sample t-test

Chi-square (*X*^2^) test was used, unless otherwise specified. Bold values indicate statistical significance *p* < 0.05

The only two factors that demonstrated significant associations with FDR cascade testing uptake across all cohorts were FDR age and FDR relationship to the proband. Across all cohorts, FDRs who underwent cascade testing were significantly younger than those who did not (total cohort: mean age 38.9 vs 50.3 years; *t*, *P* *<* 0.001). Overall uptake was significantly higher for offspring of probands than parents or siblings (24.4% vs 17.0% vs 9.3%; *χ*^2^, *P* *<* 0.001). A similar pattern was observed within the tier 1 (21.7% vs 8.1% vs 10.4%; *χ*^2^, *P* = 0.008) and emerging evidence (28.6% vs 11.1% vs 1.0%; *χ*^2^, *P* < 0.001) cohorts. In contrast, uptake within the syndromic cohort was similar among offspring and parents, yet significantly differed from that of siblings (27.7% vs 27.9% vs 13.1%; *χ*^2^, *P* *=* 0.018).

For the remaining factors, significant associations were observed for certain cohorts. Female FDRs were significantly more likely to undergo cascade testing from tier 1 (15.9% *vs* 8.6%; χ^2^, *P* *=* 0.019) and total (16.4% vs 10.4%; *χ*^2^, *P* *=* 0.012) cohorts. Symptomatic FDRs were significantly more likely to undergo cascade testing from syndromic (40.6% vs 16.5%; *χ*^2^, *P* *=* 0.001) and total (20.0% vs 12.6%; *χ*^2^, *P* *=* 0.034) cohorts. The FDRs were significantly more likely to undergo cascade testing if their proband relative was younger from syndromic (mean age 27.6 vs 42.5 years; *t*, *P* *<* 0.001) and total (mean age 42.5 vs 50.3 years; *t*, *P* *<* 0.001) cohorts.

### Impact of subsidies on cascade testing uptake

There was a significant difference in overall cascade testing uptake between FDRs who had free testing compared with those who paid out-of-pocket (21.6% vs 6.1%; *χ*^2^, *P* *<* 0.001) (Table 2). This also reached significance for tier 1 (20.1% vs 4.8%; *χ*^2^, *P* *<* 0.001) and syndromic (43.4% vs 7.1%; *χ*^2^, *P* *<* 0.001) cohorts, but not for the emerging evidence cohort (5.4% vs 9.3%; Fisher’s Exact Test [FET], *P* *=* 0.465).

For FDRs tested through the laboratory with the time limited subsidy, the mean duration from the report date of the proband’s genetic test result to FDR testing was significantly shorter for FDRs who had free testing compared to those who paid out-of-pocket (mean days 83.1 vs 212.6; *t*, *P* *=* 0.001) (Table 3).

**Table 3:** Duration from the report date of the proband’s genetic result to FDR testing

Variable	Category	Mean (CI)	*P*-value^a^
Duration to FDR testing (days)	Free	83.1 (65.3–100.9)	**0.001**
	Not free	212.6 (140.9–284.2)	

*CI* confidence interval (95%), *FDR* first-degree relative

^a^Independent sample *t*-test

Statistically significant *P*-values (<0.05) are shown in bold

As eligibility for the LKCNCCS subsidy could not be ascertained for FDRs who did not undergo cascade testing, proband eligibility was used as a proxy for FDR eligibility as socio-economic status is often similar within families. Significance was only observed for the syndromic cohort, where FDRs were more likely to undergo cascade testing if their proband relative was eligible for the LKCNCCS subsidy (28.0% vs 14.5%; *χ*^2^, *P* *=* 0.011) (Table 2).

To further evaluate the impact of the LKCNCCS subsidies on those with financial difficulties, the proportions of tested FDRs based on subsidy eligibility were compared (Table 4). For FDRs who qualified for the LKCNCCS subsidy, there was no significant difference between those who were eligible and were not eligible for free testing provided by the laboratory (52.2% vs 47.8%; *χ*^2^, *P* = 0.835). However, among tested FDRs who did not qualify for the LKCNCCS subsidy, the proportion of those undergoing free testing provided by the laboratory was significantly higher than those paying out-of-pocket (68.5% vs 31.5%; *χ*^2^, *P* < 0.001).

**Table 4:** Proportion of FDRs accessing laboratory-provided free testing, by LKCNCCS subsidy eligibility

Variable	Category	*n* (%)	*P*-value^a^
*LKCNCCS subsidy*	Free testing by laboratory	12 (52.2)	0.835
	Not free testing by laboratory	11 (47.8)	
*No LKCNCCS subsidy*	Free testing by laboratory	61 (68.5)	**0.001**
	Not free testing by laboratory	28 (31.5)	

*FDR* first-degree relative, *LKCNCCS* Lee Kong Chian National Cancer Centre Singapore Cancer Genetics Service fund

^a^Chi-square (*χ*^2^) test

Statistically significant *P*-values (<0.05) are shown in bold

## Discussion

This study reports on factors significantly associated with cascade testing uptake amongst FDRs of probands found to carry a PV/LPV in a gene associated with cancer predisposition. The findings provide valuable insights into the factors that may act as barriers or facilitators of cascade testing amongst at-risk relatives and identify specific groups where targeted interventions may help improve uptake in Singapore.

Consistent with our previous research of probands,^17^ offering subsidized cascade testing to FDRs significantly increases uptake. The uptake rate of cascade testing more than tripled when free testing was available. This is concordant with findings from other groups both within Singapore^23^ and internationally.^32^ These findings, however, are in contrast to those reported among the Malaysian population.^22^ Despite the availability of free genetic counseling and cascade testing to at-risk relatives belonging to families with a *BRCA1* or *BRCA2* PV/LPV, Yoon et al. reported an 11.0% uptake rate amongst at-risk relatives. Amongst FDRs who underwent free cascade testing in our tier 1 cohort, which includes those belonging to families with a *BRCA1/2* PV/LPV, the uptake rate was 20.1%. Malaysia and Singapore are geographical neighbours and although they are comprised of three similar ethnic groups, their majority populations differ—Chinese (74.3%) in Singapore and Malays (67.3%) in Malaysia.^31,33^ As we observed no significant differences in uptake between ethnic groups in the tier 1 cohort, it is likely that other country-specific cultural and social factors, as well as differences in service delivery, are influencing the decision-making of at-risk relatives to proceed with cascade testing. This highlights the importance of reporting on country specific data in order to appropriately guide health policy.

The provision of free cascade testing observed the most profound increase in uptake in the syndromic cohort, where FDRs were six times more likely to undergo testing when cost was removed. The majority of genetic conditions within this group have pediatric onset, where cascade testing is offered to children. Given the typical age of onset associated with these conditions, newly identified families are younger on average, with children or young parents as probands. As such, the decision to proceed with cascade testing for the majority of FDRs belonging to each family within the syndromic group is made by the same parent(s) who is already well informed as a result of prior genetic counseling. Exposure to genetic counseling, and thus improved comprehension of complex genetic risk information, is likely a facilitator of testing uptake among at-risk relatives. Patient preferences regarding novel methods for notifying and educating at-risk relatives about cascade testing, such as use of multimedia and interactive technology, should be explored.

In contrast to a recent USA study,^32^ less than one-tenth of FDRs in the emerging evidence cohort underwent testing and uptake remained low despite the availability of free testing. Interestingly, previous research demonstrated a preference among Singaporean breast and ovarian cancer probands for broad testing encompassing emerging evidence genes.^34^ This does not appear to translate to higher testing uptake amongst FDRs, either due to reluctance amongst probands to share these results, or unwillingness amongst FDRs to undergo cascade testing for less well-characterized genes.

Given the laboratory-imposed time limit on free testing, it was expected that demand for genetic counseling services within the timeframe would increase, as illustrated by the significant reduction in duration to FDR testing. Although beneficial, this added considerable pressure on clinical resources and the addition of a weekly ‘cascade testing’ clinic was required. This often resulted in multiple family members attending appointments within the same clinic. Empirical studies demonstrate that medical decision-making in Eastern cultures is commonly approached with significant family input.^22,35^ This approach stems from the concept that illness is a family event, rather than an individual experience, and involvement of the family allows for the provision of support, strength, and hope.^36,37^ This pattern of family decision-making is reflected in how families presented for cascade testing in our cohort. Whilst only a third of families had at least one FDR attend for cascade testing, more than two-thirds of these families who underwent cascade testing exceeded the cost-effectiveness threshold of 36.0% estimated by Li et al.^17^ Furthermore, the uptake rate amongst FDRs was 100.0% for approximately a quarter of all tested families. Our data demonstrates that cascade testing clusters in families, thus suggesting a family decision-making approach to genetic testing in Singapore. However, this does raise concerns of family coercion^22^ and poses ethical challenges to the genetic counseling process, such as respecting patient autonomy. Further exploration of patient experiences and preferences are needed regarding family-based genetic counseling in this setting.

In addition to resource constraints, time restrictions on free testing raise ethical considerations. Whilst this has led to greater access, there is potential for increased pressure on individuals to proceed with cascade testing within the designated time period. Decision-making regarding testing can be complex and timing is an important consideration, especially when involving children^38^ and during times of family crisis. If individuals prioritize cost, deliberative decision-making may be rushed and could translate into poorer decisional quality.^39^ Conversely, those who prioritize decision-making may end up foregoing cascade testing once cost becomes a barrier again. Furthermore, proband-mediated communication can sometimes occur years after they receive their result,^20^ thereby limiting the opportunity for all at-risk relatives to benefit from free testing. It would be prudent to consider these factors when implementing long-term funding for cascade testing programs.

Female FDRs in the tier 1 and total cohorts were significantly more likely to undergo cascade testing and is consistent with previous research, particularly for families with a *BRCA1/2* PV/LPV.^15,20,32^ The significance of younger age and relationship type to proband suggests a greater awareness of and willingness to undergo genetic testing amongst younger generations. The underrepresentation of siblings and parents may represent a tendency for probands to preferentially notify their offspring, although the underlying cause requires further investigation. Collectively, these findings illuminate groups where targeted interventions may assist in improving cascade testing uptake, including those aimed at male and older relatives.^1^

Whilst cost had a significant impact on cascade testing uptake, the overall rate was still suboptimal according to previous estimations of cost-effectiveness.^17^ Strikingly, more than two-thirds of probands have had no FDRs undergo cascade testing. It is likely that a significant number of relatives were not notified of the result. Indeed, an in-depth interview study of Singaporean women who had undergone genetic testing found multiple factors that influence result disclosure, including family closeness, perceived burden of the results on relatives, and perception of relatives’ acceptance of the result.^27^ This is particularly problematic if patients are not accurately assessing their relatives’ ability to cope with the information and their willingness to undergo testing. The challenges of proband-mediated disclosure are not unique to Singapore, however, with literature elsewhere demonstrating its ineffectiveness.^40,41^ In contrast, health professionals directly contacting at-risk relatives with proband consent has been shown to result in dramatic improvements in cascade testing uptake and is psychologically safe.^40,42,43^ Whether this is a feasible and acceptable approach in Singapore remains to be further explored. In the meantime, alternative approaches supporting proband-mediated disclosure should be considered, such as newer web-based family communication tools.^44,45^ Furthermore, the lack of protective legislation against genetic discrimination in certain countries, including Singapore, has been demonstrated to impede family communication and testing uptake.^22,23,30^ As genetic and genomic testing is becoming increasingly commonplace in all areas of medicine, it would be prudent to address these issues to ensure the progress of genomic medicine is not hindered in Singapore.

The prospective and reliable nature of the data presented strengthens the findings of this study. However, there are a number of factors potentially associated with uptake that have not been investigated, such as education level. Additionally, some data were not able to be collected for FDRs who did not undergo testing, such as their financial status. The uptake rate may be underestimated as any FDRs who had undergone testing elsewhere were unknown. Untested FDRs who had exceeded the three-month time limit were recorded as having been eligible for free testing provided by the laboratory, likely resulting in an underestimation of the association of cost with uptake. Longer follow-up is required to appreciate any differences for smaller subgroups.

In this study of cascade testing for cancer predisposition, the removal of cost as a barrier resulted in a significant increase in uptake. Current subsidy sources are not ideal, as they are either finite or impose time restrictions on families that have the potential to cause additional pressure to undergo testing. Other factors influencing cascade testing uptake should be addressed with specific interventions, such as those targeting underrepresented age and gender groups. Uptake clusters within families, therefore highlighting the need to develop and enhance genetic counseling approaches to support family communication and decision-making, whilst balancing respect for autonomy and privacy. Further research is imperative to explore alternative means for dissemination of results, including use of web-based family communication tools and the feasibility and acceptability of health professional-mediated results disclosure in Singapore.

## Methods

### Subjects

Probands who had undergone genetic testing through the CGS and were found to carry a PV/LPV in a gene associated with cancer predisposition were identified. Probands with PV/LPV(s) in genes associated with *only* autosomal recessive conditions were excluded (e.g. *MUTYH*). Families were included in the study if the proband: (i) was a Singaporean citizen or permanent resident, (ii) resided in Singapore, and (iii) had received their genetic result between November 2014 and February 2019. The probands’ FDRs were excluded from the study if they were: (i) deceased, (ii) known to reside outside of Singapore, and (iii) under the Singapore age of majority (21 years) if they belonged to a family with an adult-onset genetic condition. For probands with apparently mosaic findings, only offspring were considered as eligible FDRs. Written informed consent for medical record research was obtained from probands and FDRs at the point of genetic testing. The study was approved by the SingHealth Centralised Institutional Review Board (CIRB number is 2011/826/B).

The CGS approach to proband testing was described in detail previously.^34^ Following the identification of a PV/LPV in a proband, family notification letters were provided to assist with proband-mediated dissemination of the result. Relatives were then required to obtain a referral to the CGS from either a general practitioner or other medical specialist. An appointment for genetic counseling was scheduled upon referral. Follow-up appointments for probands were typically scheduled 6–12 months following results disclosure, however, this varied depending on the patient’s health status and whether they were on active treatment. Genetic counseling services at the CGS were provided by a cancer geneticist and/or Master’s-trained genetic counselors.

### Cascade testing subsidies

At-risk relatives who met certain financial criteria in accordance with other Singaporean government health subsidy schemes (Community Health Assist Scheme, CHAS, and Medifund)^46^ were eligible for free cascade testing via subsidies provided by the LKCNCCS fund.

During the study period, two clinical genetic testing laboratories introduced free cascade testing for FDRs of tested probands with a PV/LPV detected in the respective laboratory. Both laboratories were located in the USA and certified under the Clinical Laboratory Improvement Amendments (CLIA). For one laboratory, the free testing was only available to FDRs for a period of three months from the proband’s result report date.

For FDRs who did not meet either criterion, cascade testing carried a mean out-of-pocket cost of approximately USD 450, as genetic testing is not covered by healthcare insurance policies and/or government subsidies in Singapore.

Genetic counseling consultations incurred an out-of-pocket cost for all patients, however, were part-subsidized by the Singapore government.^46^

### Data collection

Data collection was performed prospectively during the period of November 2014 to May 2019. Demographic and clinical data were gathered from the CGS database (REDCap Software, Version 6.10.3, 2017, Vanderbilt University) for eligible probands and FDRs. Demographic and clinical data for untested FDRs were ascertained from pedigrees provided by probands. For all probands and FDRs, the age recorded on proband pedigrees was used. Subsidy eligibility and uptake of cascade testing were recorded for all FDRs. As financial status was only known for FDRs who attended the CGS, FDRs who did not undergo cascade testing were assumed to be ineligible for the LKCNCCS subsidy. Those FDRs who did not undergo cascade testing and had exceeded the three-month time limit were recorded as having been eligible for free testing provided by the laboratory. The proband’s result report date and date of FDR testing was recorded.

### Data analysis

Factors potentially prognostic for cascade testing uptake were compared between tested and untested FDR subjects. For demographic and clinical factors specific to FDRs and probands, analysis was performed overall and within three groups categorized by gene type. Genes classified as *tier 1* included *BRCA1*, *BRCA2*, and the mismatch repair genes; genes classified as *syndromic* included those associated with other well-characterized hereditary cancer syndromes (e.g. *APC*, *PTEN*, and *TP53*); and genes classified as *emerging evidence* included those that are less well-characterized and have uncertain or unknown cancer penetrance estimates (see Supplementary Table 1 for genes included in each cohort). Mean duration from the proband’s genetic result report date to FDR testing was compared before and after the availability of free testing via the laboratory. A chi-square (*χ*^2^) test and an independent sample *t*-test (both two-tailed) were used for categorical and normally-distributed continuous variables, respectively. For categorical variables (2 × 2 tables), a two-tailed FET was used when the expected count was less than 5. Statistical significance was set at *P* < 0.05. All statistical analyses were performed using IBM SPSS® version 25.

### Reporting summary

Further information on research design is available in the Nature Research Reporting Summary linked to this article.

## Supplementary information

Supplementary Table 1.

Reporting Summary Checklist

## Data Availability

The authors declare that all data supporting the findings of this study are available within the paper and its Supplementary Information file.

## Supplementary information

**Supplementary information** accompanies the paper on the *npj Genomic Medicine* website (10.1038/s41525-019-0096-5).
