# Supplementary material for: Impact of free cancer predisposition cascade genetic testing on uptake in Singapore
Source: NPJ Genom Med. 2019 Sep 13;4:22. doi: 10.1038/s41525-019-0096-5 (PMC6744424; doi:10.1038/s41525-019-0096-5)
Supplement: Supplementary file 1 — Supplementary Table 1. [file 41525_2019_96_MOESM1_ESM.pdf]

SUPPLEMENTARY TABLES

Supplementary Table 1. Genes included in each cohort

Tier 1	Syndromic	Emerging Evidence
BRCA1	*APC*	*ATM*
BRCA2	*CDH1*	*BARD1*
MLH1	*CDKN2A*	*BRIP1*
MSH2	*GATA1*	*CHEK2*
MSH6	*MEN1*	*MAX*
PMS2	*NF1*	*NBN*
	PTEN	*PALB2*
	RB1	*RAD51D*
	RET	
	SDHA	
	SDHB	
	SDHD	
	SMARCB1	
	STK11	
	TERC	
	TERT	
	TP53	
	TSC1	
	TSC2	
	VHL	
